# Short-term effects and economic burden of air pollutants on acute lower respiratory tract infections in children in Southwest China: a time-series study

**DOI:** 10.1186/s12940-023-00962-3

**Published:** 2023-01-14

**Authors:** Yi He, Wanyanhan Jiang, Xi Gao, Chengwei Lin, Jia Li, Lian Yang

**Affiliations:** 1 HEOA Group, School of Public Health, Chengdu University of Traditional Chinese Medicine, No. 1166 Liutai Road, Chengdu, China; 2 HEOA Group, School of Management, Chengdu University of Traditional Chinese Medicine, No. 1166 Liutai Road, Chengdu, China

**Keywords:** Acute lower respiratory tract infections, Children, Economic cost, Hospital admissions, Air pollutants

## Abstract

**Background:**

There are few studies on the effects of air pollutants on acute lower respiratory tract infections (ALRI) in children. Here, we investigated the relationship of fine particulate matter (PM_2.5_), inhalable particulate matter (PM_10_), sulfur dioxide (SO_2_), and nitrogen dioxide (NO_2_) with the daily number of hospitalizations for ALRI in children in Sichuan Province, China, and to estimate the economic burden of disease due to exposure to air pollutants.

**Methods:**

We collected records of 192,079 cases of childhood ALRI hospitalization between January 1, 2017 and December 31, 2018 from nine municipal/prefecture medical institutions as well as the simultaneous meteorological and air pollution data from 183 monitoring sites in Sichuan Province. A time series-generalized additive model was used to analyze exposure responses and lagged effects while assessing the economic burden caused by air pollutant exposure after controlling for long-term trends, seasonality, day of the week, and meteorological factors.

**Results:**

Our single-pollutant model shows that for each 10 μg/m^3^ increase in air pollutant concentration (1 μg/m^3^ for SO_2_), the effect estimates of PM_2.5_, PM_10_, SO_2_, and NO_2_ for pneumonia reached their maximum at lag4, lag010, lag010, and lag07, respectively, with relative risk (RR) values of 1.0064 (95% CI, 1.0004–1.0124), 1.0168(95% CI 1.0089–1.0248), 1.0278 (95% CI 1.0157–1.0400), and 1.0378 (95% CI, 1.0072–1.0692). By contrast, the effect estimates of PM_2.5_, PM_10_, SO_2_, and NO_2_ for bronchitis all reached their maximum at lag010, with RRs of 1.0133 (95% CI 1.0025–1.0242), 1.0161(95% CI 1.0085–1.0238), 1.0135 (95% CI 1.0025–1.0247), and 1.1133(95% CI 1.0739–1.1541). In addition, children aged 5–14 years were more vulnerable to air pollutants than those aged 0–4 years (*p* < 0.05). According to the World Health Organization’s air quality guidelines, the number of ALRI hospitalizations attributed to PM_2.5_, PM_10_, and NO_2_ pollution during the study period was 7551, 10,151, and 7575, respectively, while the incurring economic burden was CNY 2847.06, 3827.27, and 2855.91 million.

**Conclusion:**

This study shows that in Sichuan Province, elevated daily average concentrations of four air pollutants lead to increases in numbers of childhood ALRI hospitalizations and cause a serious economic burden.

**Supplementary Information:**

The online version contains supplementary material available at 10.1186/s12940-023-00962-3.

## Background

Air pollution is a major environmental hazard as well as a leading cause of morbidity and mortality worldwide. The number of deaths caused by air pollution has substantially increased globally over the past 20 years, with ambient air pollution causing 2.9 million deaths in 2000, 4.2 million deaths in 2015, and 4.5 million deaths in 2019 [1]. Children are an especially vulnerable group, and a large body of research has shown that air pollution is associated with poor birth outcomes and respiratory diseases in children and poor mental health in adulthood [2–6]. Acute lower respiratory tract infections (ALRI), including lung and alveolar (pneumonia) as well as airway infections (bronchitis and bronchiolitis), are the leading cause of death in children under the age of 5 [7]. In the Global Burden of Diseases, Injuries, and Risk Factors Study 2019, lower respiratory tract infections ranked second among the top 10 causes of disability-adjusted life years in children under the age of 10 [8]. The World Health Organization (WHO) reports that approximately 93% of children under the age of 15 worldwide suffer from air pollution on a daily basis and that in 2016, 600,000 children died from ALRI caused by air pollution [9].

Children are particularly vulnerable to air pollution because their lung immune system is still developing. A number of studies conducted in different countries have demonstrated an association between long-term or short-term exposure to air pollutants and childhood ALRI hospitalization [10–13]. There are also several studies from China that have focused on the effects of air pollutants on the hospitalization of children with ALRI. For examples, a study conducted in 25 Chinese cities found that each 10 μg/m^3^ increase in the concentrations of the gaseous pollutants SO_2_ and NO_2_ resulted in an increase of 0.54% (95% CI, 0.30–0.79) and 0.60% (95% CI, 0.22–0.99) in respiratory hospitalizations among children aged 0–14 years, respectively [14]. Yang et al. investigated the association between short-term exposure to air pollutants and hospitalizations for ALRI in children aged 0–14 years in four Chinese cities, Guangzhou, Shanghai, Wuhan, and Xining [15]. Another study conducted in Shanghai found that with each interquartile range (IQR) increase in PM_2.5_, emergency department visits for pediatric bronchitis and pneumonia increased by 1.53% (95% CI, 0.01–3.08) and 1.90% (95% CI, 0.30–3.52) on lag3, respectively [16]. Moreover, a study conducted in Guangzhou by Liang et al. made a similar conclusion that an elevation in particulate matter concentrations is significantly associated with an increase in outpatient visits for pneumonia and bronchitis in children [17]. However, the above studies were conducted mostly in central and eastern parts of China, while there are relatively few studies on the effects of air pollution on childhood ALRI hospitalization in the western regions; moreover, most of the existing ones are single-city studies [18–20]. It has also been shown that children in the western regions of China are most vulnerable to air pollution [21]. Sichuan Province, which constitutes the largest economy in western China, is located in the southwest and has approximately 90 million inhabitants [22]. In terms of topography, it is roughly divided into the Sichuan Basin and the Western Sichuan Plateau. As the degradation of pollutants is subject to geographical factors, the alpine terrain of the basin and surrounding plateaus causes relatively slow diffusion of atmospheric pollutants, making the Sichuan Basin the fourth most polluted area, after the Beijing-Tianjin-Hebei region, the Yangtze River Delta, and the Pearl River Delta [23]. In a study conducted in Sichuan by Pu et al., only the effects of particulate matter were analyzed and the health effects of common air pollutants on lower respiratory tract diseases in children were not fully reflected [24]. Here, we therefore performed a time-series analysis to more comprehensively assess the effects of short-term exposure to PM_2.5_, PM_10_, SO_2_, and NO_2_ on childhood hospitalization for ALRI (including pneumonia and bronchitis) based on hospitalization data of children aged 0–14 years from urban medical institutions in nine cities and prefectures of Sichuan Province between 2017 and 2018. This study provides more scientific basis for a comprehensive evaluation of the impact of air pollution on children’s health across different regions. Moreover, we analyzed the effects of sex, age, and season, while estimating the economic burden of diseases due to exposure to air pollutants.

## Methods

### Data on ALRI in children

Sichuan Province comprises a total of 21 cities and prefectures, including 18 in the Sichuan Basin and three in the western Sichuan Plateau. For this study, we collected data on a total of 192,079 cases of pediatric ALRI inpatients aged 14 years and younger from January 1, 2017 to December 31, 2018, including each child’s age, sex, home address, admission date, discharge date, disease diagnosis, disease code, and total hospitalization cost, from nine city or prefecture hospitals in Chengdu, Guang’an, Luzhou, Liangshan Yi Autonomous Prefecture, Mianyang, Meishan, Nanchong, Yibin, and Zigong of Sichuan Province. The causes of hospital visits were coded according to the International Classification of Diseases, 10th Revision (ICD-10), as follows: pneumonia (J12–J18), bronchitis (J20–J21), and other acute lower respiratory tract infections (J22). The latter were not analyzed separately because we counted only nine children with such infections in the entire sample. Of the above nine cities and prefectures, eight are located in the Sichuan Basin and one in the Western Sichuan Plateau. Our samples therefore represent, to a certain extent, the overall situation in Sichuan Province.

### Meteorological and pollutant data

We collected daily meteorological data from the Sichuan Meteorological Bureau (http://www.scdata.net.cn/) between January 1, 2017 and December 31, 2018, including daily average temperature and relative humidity. Particulate matter with an aerodynamic diameter ≤ 10 μm (PM_10_), particulate matter with an aerodynamic diameter ≤ 2.5 μm (PM_2.5_), sulfur dioxide (SO_2_), and nitrogen dioxide (NO_2_) were chosen as indicators of outdoor air pollution, as these four pollutants are closely associated with respiratory diseases. The daily average concentrations of PM_2.5_, PM_10_, SO_2_, and NO_2_ were collected from environmental monitoring stations in Sichuan Province (including a total of 183 air monitoring stations) during the same period.

Air pollutant exposure was assessed by using the inverse distance weighting (IDW) method. Specifically, the locations of all monitoring stations and home addresses of hospitalized cases of pediatric ALRI were geo-coded using the Gaudet Map API (https://lbs.amap.com/). For each hospitalized case and monitoring station, the inverse distance (1/distance^2^) weighted average of the concentrations from all monitoring stations was used to estimate air pollutant exposure on the hospitalization day (lag0), single-day lags from the current day (lag0) and each 1–10 days before the ALR events (lag1, lag2, lag3, lag4, lag5, lag6, lag7, lag8, lag9, and lag10), as well as multi-day moving average lag exposures (lag01, lag02, lag03, lag04, lag05, lag06, lag07, lag08, lag09, and lag010).

### Health efects of air pollutants exposure in overall and subgroup

populationThe correlation between air pollutants and meteorological indicators was analyzed using the Spearman correlation test, with absolute values of the correlation coefficient r closer to 1 indicating a stronger correlation.

Time series analysis methods applied to the generalized additive model (GAM) have been repeatedly used to assess the association between air pollutants and hospitalizations for respiratory diseases. Since the daily number of hospitalizations for pediatric ALRI usually obeys an over-dispersed Poisson distribution, a quasi-Poisson GAM model was adopted in this study. As mentioned above, our analysis of the correlations of PM_2.5_, PM_10_, SO_2_, and NO_2_ with the daily number of hospitalizations was based on single-day lags exposures (lag1, lag2, lag3, lag4, lag5, lag6, lag7, lag8, lag9, and lag10) and multi-day moving average lags exposures (lag01, lag02, lag03, lag04, lag05, lag06, lag07, lag08, lag09, and lag010). Daily average temperature and relative humidity were introduced into the model as control variables. The GAM model was formulated as follows:

1 $${\displaystyle \begin{array}{l} Log\left[E(Yi)\right]=\alpha +\beta Zi+s\left( time,k= df+1\right)+\textrm{s}\ \left(\textrm{temperature},\textrm{k}=\textrm{df}+1\right)\ \\ {}\kern5.5em +\textrm{s}\ \left(\textrm{humidity},\textrm{k}=\textrm{df}+1\right)+\textrm{as}.\textrm{factor}\left(\textrm{dow}\right)\end{array}}$$

where *E (Yi)* is the expected number of children hospitalized for ALRI on day *i*; *α* stands for the model intercept; *Zi* represents the air pollutant concentration on day *i* (μg/m3); *β* represents the exposure - response coefcient, which is the increase in the number of daily hospitalizations caused by the per unit increase in pollutant concentration; *s* is a non-smooth parameter item; and *df* is the degree of freedom. The time stands for a date variable with a degree of freedom of 7/year; dow is an indicator variable of “day of the week”; temperature and humidity stand for the average daily temperature and relative humidity, whose degrees of freedom are both 3.

The exposure-response coefficient *β* was estimated by the generalized additive model (Eq. 1). Subsequently, the relative risk (RR) and 95% CI of children hospitalized with ALRI for every 10 μg/m^3^ (1μg/m^3^ for SO_2_) increase in the concentration of air pollutants were calculated.

To identify potentially susceptible populations, we performed stratified analyses according to sex (boys and girls), age (0–1 years, 2–4 years, and 5–14 years), and season (hot season, June to August; transitional season, April, May, September, and October; cold season, November to March). The 95% confidence interval (95% CI) for the difference in the effect estimates between different categories in each subgroup (e.g., boys and girls) was calculated by the following formula to test whether a difference was statistically significant:

2 $$\left(\hat{Q}1-\hat{Q}2\right)\pm \sqrt{{\left(S\hat{E}1\right)}^2+{\left(S\hat{E}2\right)}^2}$$

where $\hat{Q}1$ and $\hat{Q}2$ are the estimates of different categories in each subgroup, and $S\hat{E}1$ and $S\hat{E}2$ are the corresponding standard errors for each estimate [25].

### Attributable health risks and economic costs due to air pollution

The attributable number of ALRI hospitalizations due to air pollutants exposure was calculated according to the attributable risk equation [26]. The air pollutant concentrations in the air quality guidelines issued by the WHO were used as a standard reference (24-hour average: 15 μg/m^3^ for PM_2.5_, 45 μg/m^3^ for PM_10_, 40 μg/m^3^ for SO_2_, and 25 μg/m^3^ for NO_2_) [27]. The attribution eqs. 3, 4, and 5 were formulated as follows:

3 $${\textrm{AN}}_{\textrm{i}}=\left(\exp \left(\upbeta \ast {\Delta \textrm{AP}}_{\textrm{i}}\right)\hbox{-} 1\right)/\exp \left(\upbeta \ast {\Delta \textrm{AP}}_{\textrm{i}}\right)\ast {\textrm{N}}_{\textrm{i}}\kern1em$$

4 $$meanC={c}_h+\textrm{dPCDI}\times {\textrm{meanT}}_{\textrm{h}}$$

5 $$\varDelta C= AN\times meanC$$

In these equations, *AN*_*i*_ is the number of hospitalizations attributed to air pollutants exposure on day *i*, *β* is the exposure-response coefficient (Eq. 1) between pollutants and the number of ALRI hospitalizations, *ΔAP*_*i*_ is the difference between the observed and reference concentrations of air pollutants on day *i*, *N*_*i*_ is the number of ALRI hospitalizations on day *i*, *meanC* is the average total economic cost of hospitalization per case, *c*_*h*_ is the average total hospitalization cost per case during the study period, *dPCDI* is the per capita daily disposable income in Sichuan Province, *meanT*_*h*_ is the average number of hospitalization days per case, and *ΔC* is the total economic cost attributed to air pollution.

All statistical analyses in this study were carried out using R3.4.3, and the quasi-Poisson regression model was constructed using the package “mgcv”. The test level was α = 0.05.

### Sensitivity analysis

Three types of sensitivity analyses were performed to verify the stability of the model. First, if a significant association between a given air pollutant and childhood ALRI was observed, a two-pollutant models was further fitted to assess the robustness of our results. Second, the stability of the time trend was tested and the model was fitted by varying the degrees of freedom of the time series (df = 5, 6, 8, 9/year). Finally, a sensitivity analysis was performed by assessing the number of ALRI cases within a circular 50 km area around the air monitoring station. By doing this we were able to assess the potential impact of the distance between the air pollution monitoring site and the patient’s home address [28].

## Results

Table 1 provides air pollution levels, meteorological variables, and daily ALRI hospitalizations. The daily average concentrations of PM_2.5_, PM_10_, SO_2_, and NO_2_ during the study period were 46.01, 71, 12.01, and 28.93 μg/m^3^, respectively. The daily average concentrations of PM_2.5_, PM_10_, and NO_2_ exceeded the standards of the air quality guidelines issued by the WHO (15, 45, and 25 μg/m^3^) [27], and the numbers of days on which the concentrations of PM_2.5_, PM_10_, and NO_2_ exceeded the standards were 698, 497, and 439, respectively. The daily average temperature and relative humidity were 17.81 °C and 76.82%. A total of 192,079 hospitalized cases of childhood ALRI were enrolled during the study period, including 129,870 cases of pneumonia and 62,200 of bronchitis. The average daily number of hospitalizations was higher in boys than in girls, and the age group from 0 to 1 years displayed the highest average daily hospitalizations (111 cases). Regarding the three seasons we defined for our analyses, the highest average daily number of hospitalizations (336 cases) was recorded during the cold season. Moreover, the daily number of hospitalizations for pneumonia was larger than that for bronchitis.

**Table 1:** Description of daily air pollutants, meteorological parameters, and hospitalized characteristics of children with ALRI in the Sichuan Province, China, during 2017–2018

stats	mean ± sd	min	max	p25	p50	p75
Air pollutant concentration						
PM_2.5_	46.01 ± 30.34	9.92	178.73	24.55	36.68	59.49
PM_10_	71 ± 41.05	18.49	226.57	39.91	59.42	93.79
SO_2_	12.01 ± 3.11	6.65	26.76	9.67	11.38	13.59
NO_2_	28.93 ± 8.36	14.03	59.09	22.4	27.51	34.86
Meteorological measures						
Temperature(°C)	17.81 ± 7.29	2.9	30.98	10.73	17.74	24
Humidity(%)	76.82 ± 9.55	49.31	94.83	70.54	77.85	84.11
Hospital admissions(N)	263.12 ± 102.36	104	817	191	247.5	310
Sex(N)						
Boys	149 ± 59	54	468	109	141	175
Girls	114 ± 46	37	349	81	106	135
Age(N)						
0-1 years	111 ± 49	36	286	75	98	133
2-4 years	103 ± 43	26	315	72	98	129
5-14 years	49 ± 23	11	216	32	44	60
Season(N)						
Warm season	1689 ± 409	104	284	137.5	161	193
Transition season	245 ± 62	108	484	207	245	278
Cold season	336 ± 101	136	817	253	313	414
Acute lower respiratory infections						
Pneumonia	178 ± 74	13	539	127	166	209
Bronchitis	85 ± 33	27	278	62	81	103

*sd* standard deviation, *p25* 25th percentile, *p50* 50th percentile, *p75* 75th percentile

We observed significant positive correlations between PM_2.5_ and PM_10_, SO_2_, and NO_2_, with the correlation coefficient r ranging from 0.3465 to 0.9442. In addition, PM_10_ showed a strong positive correlation with SO_2_ and NO_2_, with correlation coefficients of 0.3428 and 0.6392, respectively. The coefficient for the correlation between SO_2_ and NO_2_ was 0.2, and our analyses revealed a negative correlation between air pollutants and meteorological variables (*p* < 0.05) (Table S1).

### Effects of pollutants on health of subgroups and overall population

**Fig. 1:**
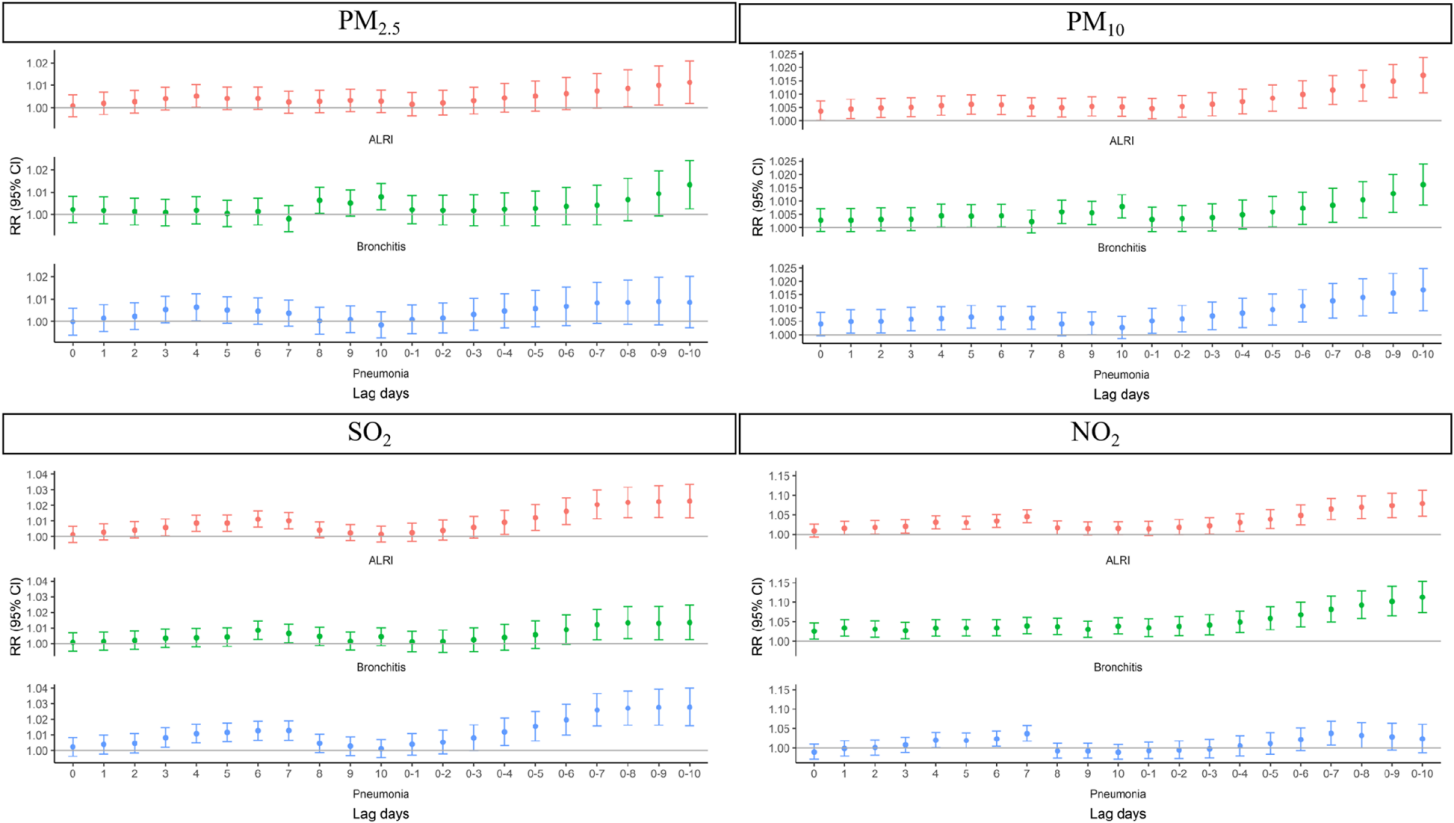
The associations between the concentrations of air pollutants and the number of children hospitalized for ALRI in the single-pollutant model in the Sichuan Province, China, during 2017–2018

Fig. 1 shows the relationship between each 10 μg/m^3^ increase in the concentrations of PM_2.5_, PM_10_, SO_2_, and NO_2_ and hospitalizations for overall ALRI, pneumonia or bronchitis, at different lag days. While the effects of PM_2.5_, PM_10_, SO_2_, and NO_2_ on hospitalizations all reached their maximum at lag010. The differences in these effects were statistically significant. For pneumonia, the effect estimates for PM_2.5_, PM_10_, SO_2_, and NO_2_ reached their maximum at lag4, lag010, lag010, and lag07, respectively, with RR values of 1.0064 (95% CI, 1.0004–1.0124), 1.0168(95% CI 1.0089–1.0248), 1.0278 (95% CI 1.0157–1.0400), and 1.0378 (95% CI, 1.0072–1.0692). For bronchitis, the differences in the effect estimates of the effect estimates of PM_2.5_, PM_10_, SO_2_, and NO_2_ all reached their maximum at lag010, with RR values of 1.0133 (95% CI 1.0025–1.0242), 1.0161(95% CI 1.0085–1.0238), 1.0135 (95% CI 1.0025–1.0247), and 1.1133(95% CI 1.0739–1.1541).

**Fig. 2:**
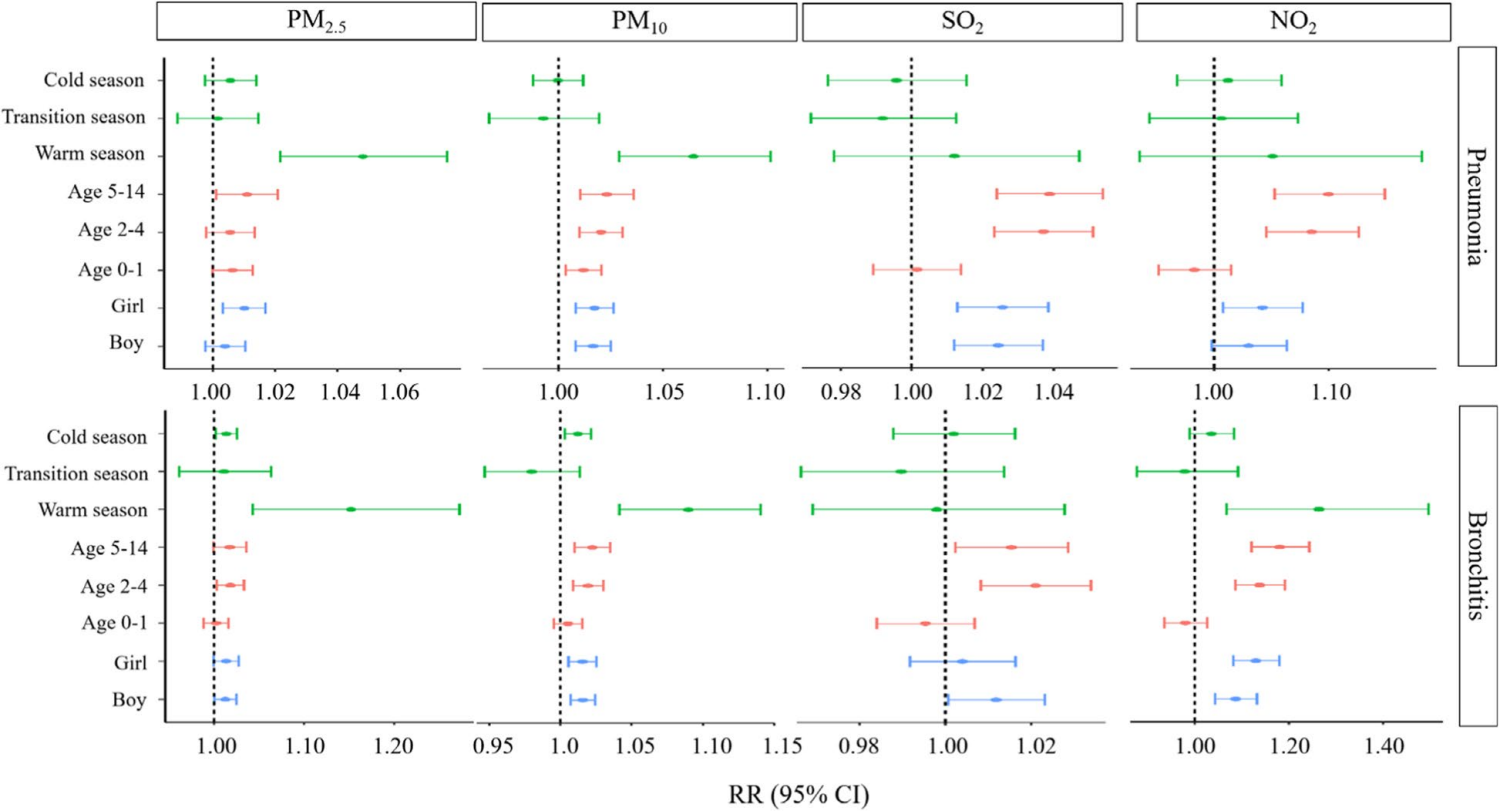
RR (95% CI) of stratified analyses for each air pollutant in the single-pollutant model in the Sichuan Province, China, during 2017–2018

Fig. 2 illustrates how each 10 μg/m^3^ increase in the concentrations of PM_2.5_, PM_10_, SO_2_, and NO_2_ affects incidences of pneumonia and bronchitis in children of different sexes and ages and in different seasons after adjusting for temperature, relative humidity. The effects of the four air pollutants were significant overall, but there were no statistically significant differences between girls and boys (*p* > 0.05). SO_2_ and NO_2_ caused significantly more hospitalizations for pneumonia or bronchitis in children aged 5–14 years than in those aged 0–1 years and 2–4 years (*p* < 0.05). Both PM_2.5_ and PM_10_ displayed a positive and significant effect on hospitalizations for pediatric pneumonia during the warm season, which statistically differed from the effects observed in the transitional and the cold season (*p* < 0.05). Similarly, PM_2.5_, PM_10_, and NO_2_ had a positive and significant effect on hospitalizations for bronchiolitis during the warm season, which statistically differed from the effects observed in the transitional and the cold season (*p* < 0.05) (Table S2).

Based on the largest effect estimates in single-pollutant model, pneumonia PM_2.5_ is lag4, PM_10_ and SO_2_ are lag010，NO_2_ is lag07; In bronchitis, PM_2.5_, PM_10_, SO_2_ and NO_2_ are lag010.

Table 2 summarizes the attributable number (AN), attributable risk (AR), and economic cost of ALRI hospitalizations related to PM_2.5_, PM_10_, and NO_2_ pollution in Sichuan Province from 2017 to 2018 using the air quality guidelines of the WHO as a reference standard. Since the daily recorded average concentration of SO_2_ at lag010 was far lower than the reference concentration (40 μg/m^3^), we did not measure the AN, AR, or economic costs related to SO_2_. According to the WHO reference concentrations, the number of ALRI hospitalizations attributable to PM_2.5_, PM_10_, and NO_2_ pollution was 7551, 10,151, and 7575, respectively. The total economic cost of ALRI attributable to PM_2.5_, PM_10_, and NO_2_ pollution during the study period was CNY 2847.06, 3827.27, and 2855.91 million, respectively. Among the specific diseases, pneumonia caused a higher disease burden than bronchitis.

**Table 2:** The attributable number, attributable risk and economic costs of hospital admissions related to air pollution using WHO’s air quality guidelines in Sichuan Province 2017–2018

	Pollutant	Lag days	AN	AR	Costs
ALRI	PM_2.5_	Lag010	7551	0.034(0.006–0.062)	2847.06
	PM_10_	Lag010	10,151	0.043(0.027–0.059)	3827.27
	NO_2_	Lag010	7575	0.029(0.018–0.041)	2855.91
Pneumonia	PM_2.5_	Lag4	2919	0.019(0.001–0.037)	1236.21
	PM_10_	Lag010	6775	0.043(0.023–0.062)	2869.56
	NO_2_	Lag07	2706	0.016(0.003–0.028)	1146.03
Bronchitis	PM_2.5_	Lag010	2913	0.041(0.008–0.073)	815.13
	PM_10_	Lag010	3111	0.040(0.022–0.059)	870.56
	NO_2_	Lag010	3031	0.034(0.023–0.045)	848.33

Based on the largest effect estimates in single-pollutant model, in ALRI and pneumonia, pneumonia PM_2.5_ is lag4, PM_10_ and SO_2_ are lag010，NO_2_ is lag07; In bronchitis, PM_2.5_, PM_10_, SO_2_ and NO_2_ are lag010.

### Sensitivity analysis

First, the correlation between particulate matter and hospitalizations for pneumonia or bronchitis weakened and became insignificant when we controlled for gaseous pollutants in the two-pollutant models. When controlling for PM_2.5_, PM_10_, and NO_2_, the association between SO_2_ and pneumonia hospitalizations alone was strong and significant. Likewise, the association between NO_2_ and bronchitis hospitalizations was strong and significant when we controlled for PM_2.5_, PM_10_, and SO_2_ **(**Table 3**)**. Second, the acute effect of air pollutants on hospitalizations did not change substantially after replacing the annual degrees of freedom of the time series with 5, 6, 8 or 9(Table S3). Finally, there were 190 patients whose home was more than 50 km away from the nearest monitoring station, including 146 with pneumonia and 44 with bronchitis. After removing the above data, the effect values for the different pollutants did not change significantly, and our observations from the sensitivity analysis did not significantly differ from the results obtained from the original model (Table S4, Table S5).

**Table 3:** RR (95% CI) of children hospital admissions per 10 μg/m^3^ increase in concentrations of pollutants in the single and two-pollutant models

Two-pollutant models		Pneumonia		Bronchitis	
		RR	95% CI	RR	95% CI
**PM**_**2.5**_	–	**1.0064**	**1.0004–1.0124**	**1.0133**	**1.0025–1.0242**
	Adjusted for SO_2_	0.9991	0.9913–1.0070	1.0091	0.9969–1.0213
	Adjusted for NO_2_	1.0040	0.9959–1.0122	0.9897	0.9765–1.0031
**PM**_**10**_	–	**1.0168**	**1.0089–1.0248**	**1.0161**	**1.0085–1.0238**
	Adjusted for SO_2_	**1.0110**	**1.0018–1.0202**	**1.0146**	**1.0064–1.0229**
	Adjusted for NO_2_	**1.0191**	**1.0099–1.0283**	1.0050	0.9959–1.0141
**SO**_**2**_	–	**1.0278**	**1.0157–1.0400**	**1.0135**	**1.0025–1.0247**
	Adjusted for PM_2.5_	**1.0324**	**1.0180–1.0471**	1.0093	0.9970–1.0218
	Adjusted for PM_10_	**1.0173**	**1.0035–1.0312**	1.0055	0.9938–1.0174
	Adjusted for NO_2_	**1.0456**	**1.0275–1.0640**	0.9900	0.9766–1.0036
**NO**_**2**_	–	**1.0378**	**1.0072–1.0692**	**1.1133**	**1.0739–1.1541**
	Adjusted for PM_2.5_	1.0346	0.9944–1.0764	**1.1379**	**1.0868–1.1913**
	Adjusted for PM_10_	1.0000	0.9640–1.0372	**1.0984**	**1.0513–1.1475**
	Adjusted for SO_2_	0.9497	0.9061–0.9954	**1.1364**	**1.0856–1.1896**

Based on the largest effect estimates in single-pollutant model, pneumonia PM_2.5_ is lag4, PM_10_ and SO_2_ are lag010，NO_2_ is lag07; In bronchitis, PM_2.5_, PM_10_, SO_2_ and NO_2_ are lag010.

## Discussion

In this study, we performed a time-series analysis to investigate the relationship between air pollutant exposure and the risk of hospitalization for ALRI in children in Sichuan Province, China, and found that short-term exposure to air pollutants increased the risk of hospitalization when data were controlled for confounders such as daily temperature and relative humidity. Stratified analyses revealed that older children were more vulnerable to outdoor air pollutants, as were all children during the hot season. Moreover, the present study identified a serious economic burden of disease due to exposure to air pollutants.

The findings of our single-pollutant model analysis are largely consistent with previously reported findings. These findings support hypothesis that short-term exposure to particulate matter is associated with ALRI hospitalizations. Specifically, it has been shown that exposure to PM_2.5_ and PM_10_ increases the risk of ALRI hospitalization in children. In this study, we observed that for each 10 μg/m^3^ increase in particulate matter concentration of PM_2.5_, an effect on pneumonia occurred only at lag4, with an RR of 1.0064(95% CI 1.0004–1.0124) and the effect on bronchitis reached its maximum at lag010 with an RR of 1.0133 (95% CI 1.0025–1.0242). By contrast, the effect of PM_10_ on both pneumonia and bronchitis reached its maximum at lag010, with RR of 1.0168 (95% CI 1.0089–1.0248)、1.0161 (95% CI 1.0085–1.0238), respectively. Multiple studies from several regions, including Taiwan and Jinan in China, found that elevated particulate matter concentrations were associated with increased outpatient visits and hospitalizations for pneumonia [29, 30]. A study conducted in South Korea revealed that the concentration of PM_2.5_ at lag06 caused the greatest risk of ALRI hospitalization in children [12]. In a case-crossover study of 112,467 children in the U.S., 1-week exposure to PM_2.5_ was found to be associated with hospitalization for ALRI in children aged 0–2 years and 3–17 years [31]. It has been demonstrated that the mechanisms underlying destructive effects of PM_2.5_ on the respiratory system involve free radical peroxidative damage, imbalance of intracellular calcium homeostasis, and inflammatory injury [32]. Besides, exposure to particulate matter has been shown to cause adverse effects on lung function in preschool children. A study from China found that for each 10 μg/m^3^ increase in the concentration of PM_2.5_, decreases in 25–75% forced expiratory flow (FEF_25–75%_), forced expiratory volume/forced vital capacity (FEV1/FVC) per second, 75% forced expiratory flow (FEF_75%_), and forced expiratory volume in 1 second (FEV1) reached their maximum at lag01, at 80.44 mL/s, 35.85%, 78.58 mL/s, and 61.34 mL, respectively [33]. Particulate pollution can also affect the spread and prevalence of respiratory viruses [34, 35]. Among these, respiratory syncytial virus (RSV) is considered to be the most important viral pathogen causing ALRI in young children [36]. A study conducted in Italy revealed that PM_10_ exposure is associated with increased hospitalizations for RSV bronchiolitis in infants, while the level of PM_10_ concentrations 2 weeks before hospital admission was closely correlated with an increased risk of hospitalization for RSV bronchiolitis [37].

We also observed that for each 1 μg/m^3^ increase in SO_2_ concentration and each 10 μg/m^3^ increase in NO_2_ concentration, the effect of SO_2_ on both pneumonia and bronchitis reached its maximum at lag010 with RRs of 1.0278 (95% CI 1.0157–1.0400), 1.0135 (95% CI 1.0025–1.0247), respectively, and the effect of NO_2_ on pneumonia reached its maximum at lag07 with an RR of 1.0378 (95% CI 1.0072–1.0692) and the effect on bronchitis reached its maximum at lag010 with an RR of 1.1133 (95% CI 1.0739–1.1541). Similarly, one study on the relationship between short-term air pollutant exposure and childhood ALRI in Nanjing, China found that for each interquartile range (IQR) increase in the concentrations of SO_2_ and NO_2_, the estimated cumulative effects of both pollutants reached their maximum at lag05 (5.6%, 2.6–8.6%; 4.1%, 1.2–7.0%) [38]. Moreover, a study in 25 cities in China revealed an association of short-term exposure to SO_2_ and NO_2_ with pneumonia and bronchitis [14]. SO_2_ is readily hydrated into sulfite (SO_3_^2−^) and bisulfate (HSO^3−^) ions, and exposure to SO_2_ can lead to mitochondrial dysfunction in the lungs, which in turn causes cellular disorders and subsequently lung diseases [39, 40]. NO_2_, a major traffic-related pollutant, has low solubility and can penetrate into the lungs [41]. NO_2_ can be absorbed throughout the respiratory tract, and incurring damage may occur in the trachea, bronchi, bronchioles, alveolar ducts, and proximal airways, depending on its concentration and dose [42, 43].

In our sex-stratified analysis, we found that while both boys and girls were vulnerable to air pollution, no significant differences were observed between the two groups. This finding is consistent with the results of other studies [44]. Meanwhile, the age-specific analysis in this study showed that SO_2_ and NO_2_ had a greater effect on hospitalizations for pneumonia and bronchitis in children aged 2–4 years and 5–14 years than in those aged 0–1 years. A multi-city study from Colombia found that both PM_2.5_ and NO_2_ had the greatest impact on emergency room visits for respiratory diseases among children aged 5–9 years [45]. Another study from China also observed that PM_2.5_, SO_2_, and NO_2_ had a greater impact on respiratory diseases in children aged 4–14 years [15]. This observation may be attributed to the fact that children aged 2–4 years and 5–14 years spend more time in school than those aged aged 0–1 years, and data shows that the average concentration of NO_2_ in the school environment exceeds the WHO guidelines [46]. Notably, an average of 82 asthma attacks per school per year could be avoided by reducing outdoor NO_2_ concentrations [47], since school-age children begin to participate in more outdoor activities and are exposed to more traffic-related pollutants. At the same time, breastfeeding improves the infant’s resistance and, with relatively quiet infants, the physical activity of larger children leads to deeper and more frequent aspiration [48, 49]. In the present study, a specific season analysis showed that compared with the transitional and the cold season, PM_2.5_ and PM_10_ had greater adverse effects on childhood pneumonia during the warm season, as had PM_2.5_, PM_10_, and NO_2_ on childhood bronchitis. Similarly, Cheng et al. found that children are more vulnerable to NO_2_ during the warm season [29]. Lv et al. also found a greater impact on child hospital admissions during the warm season [30]. The above seasonal differences could be attributed to the fact that the correlation between personal air pollutant exposure and ambient air pollutant concentrations is higher in summer than in winter, and there are differences in personal exposure between different seasons [50]. Alternatively, the concentrations of different components of particulate matter may vary among seasons [51]. The discrepancy could also be explained by the fact that high temperatures lead to more NO_2_ emissions, especially in cities with larger traffic volumes, and that higher concentrations of NO_2_ can occur in economically developed areas [52]. In this case, the spatial and temporal distribution of air pollutants could potentially be affected.

The present study not only discusses the relationship between air pollution and ALRI hospitalization in children, but also assesses the economic burden caused by ALRI hospitalizations; it therefore has important implications for improving air quality and preventing respiratory diseases. During the study period, the greatest economic burden of hospitalizations for ALRI was caused by PM_10_, followed by NO_2_, while the smallest burden was caused by PM_2.5_. Likewise, a study conducted in Guiyang, China, found that PM_10_ caused higher hospitalization costs than PM_2.5_ [53]. This finding could be explained by the fact that PM_2.5_ is included in PM_10_ concentrations, and that the two types of particulate matter are highly correlated (r = 0.9442). An earlier study that employed machine learning to verify the accuracy of linking particulate matter concentrations to upper respiratory infections found higher accuracy for PM_10_ than PM_2.5_ [54]. Overall, ALRI pose a serious burden on children and their families.

We observed that the association of PM_2.5_ with ALRI hospitalizations weakened and became insignificant after adjusting for SO_2_ and NO_2_ in our two-pollutant models. Similarly, Zheng et al. found that after controlling for SO_2_ and NO_2_, the estimates associated with PM_2.5_ in the two-pollutant models were not significant (PM_2.5_, 1.50, 95% CI, 0.35–2.66; PM_2.5_ adjusted for SO_2_, 0.17, 95% CI, − 2.55–0.43; PM_2.5_ adjusted for NO_2_, 0.17, 95% CI, − 1.47–1.85) [55]. Moreover, the authors observed that the association of SO_2_ and NO_2_ with ALRI hospitalizations became partially statistically insignificant after mutual adjustments. This observation is similar to the results of a number of previous studies [56]. There may be an interaction between SO_2_ and NO_2_; moreover, both particulate matter and NO_2_ are traffic-related pollutants and are highly correlated [44]. Thus, it is difficult to determine the individual effects of each pollutant. The above results imply that the effects of pollutants are not simply superimposed. Instead, collinearity effects between different pollutants may occur and may have a synergistic effect on the acute lower respiratory tract, thereby affecting the authenticity of the model.

The present study has the following three advantages: First, this is the first study on disease burden due to air pollutant exposure in children aged 14 years and younger in nine cities and prefectures in Sichuan Province. Second, cities and prefectures from the Sichuan Basin as well as from the western Sichuan Plateau were selected, and our sample therefore represents the overall situation in Sichuan Province. Third, the IDW interpolation method was used to construct a high spatial resolution for the estimation of pollutant concentrations, which improved the spatial accessibility of pollutants. However, this study also has some limitations. First, this is an ecological study, and our findings may be slightly inconsistent with the real situation, due to the presence of confounding factors. Second, we collected only 2 years’ worth of data for our analysis of the associations between air pollutants and hospitalizations for ALRI in children, which may have caused some instability in the model we used [57].

## Conclusion

Based on a time-series analysis, this study assessed the short-term effects of short-term air pollutant exposure on the daily number of hospitalizations for ALRI in children in Sichuan Province between 2017 and 2018, and found that elevated daily average concentrations of PM_2.5_, PM_10_, SO_2_, and NO_2_ increase hospitalizations, showing lagged effects. Moreover, we found that SO_2_ and NO_2_ have more significant effects on older children and that the economic burden due to ALRI can be partially attributed to excessive pollutant exposure. In order to protect children’s health, authorities in Sichuan Province should take effective measures to reduce the emission of harmful substances in the air.

## Supplementary Information

**Additional file 1 Table S1**. Pearson correlation coefficients between daily meteorological factors and air pollutants for Sichuan (2017–2018). **Table S2**. RR (95% CI) of stratified analyses for each air pollutant in the single-pollutant model. Based on the largest effect estimates in single-pollutant model, pneumonia PM2.5 is lag4, PM10 and SO2 are lag010，NO2 is lag07; In bronchitis, PM2.5, PM10, SO2 and NO2 are lag010. PM2.5 fine particulate matter, PM10 inhalable particulate matter, SO2 sulfur dioxide, NO2 nitrogen dioxide, CI confidence interval. **Table S3**. Association between air pollutants (10 μg/m3 increase) and the daily hospitalization in children with ALRI by degrees of freedom per year. **Table S4**. Associations between air pollutants (every 10 μg/m3 increase in the later period of retention) and hospitalization in children with pneumonia (Eliminate the data from the home address to the monitoring station greater than 50 km). **Table S5**. Associations between air pollutants (every 10 μg/m3 increase in the later period of retention) and hospitalization in children with bronchiolitis (Eliminate the data from the home address to the monitoring station greater than 50 km.

## Abbreviations

PM2.5: Particulate matter < 2.5 μm in aerodynamic diameter
SO2: Sulfur dioxide
NO2: Nitrogen dioxide
ALRI: Acute lower respiratory tract infections
IDW: Inverse distance weighting
GAM: Generalized additive model
SD: Standard deviation
RR: Relative risk
CI: Confidence interval
FEF_25–75%_: Forced expiratory flow
FEV1/FVC: Forced expiratory volume/forced vital capacity
FEF_75%_: 75% forced expiratory flow
RSV: Respiratory syncytial virus.
