# Supplementary material for: Short-term effects and economic burden of air pollutants on acute lower respiratory tract infections in children in Southwest China: a time-series study
Source: Environ Health. 2023 Jan 14;22:6. doi: 10.1186/s12940-023-00962-3 (PMC9840265; doi:10.1186/s12940-023-00962-3)
Supplement: Supplementary file 1 — Additional file 1 Table S1. Pearson correlation coefficients between daily meteorological factors and air pollutants for Sichuan (2017–2018). Table S2. RR (95% CI) of stratified analyses for each air pollutant in the single-pollutant model. Based on the largest effect estimates in single-pollutant model, pneumonia PM2.5 is lag4, PM10 and SO2 are lag010，NO2 is lag07; In bronchitis, PM2.5, PM10, SO2 and NO2 are lag010. PM2.5 fine particulate matter, PM10 inhalable particulate matter, SO2 sulfur dioxide, NO2 nitrogen dioxide, CI confidence interval. Table S3. Association between air pollutants (10 μg/m3 increase) and the daily hospitalization in children with ALRI by degrees of freedom per year. Table S4. Associations between air pollutants (every 10 μg/m3 increase in the later period of retention) and hospitalization in children with pneumonia (Eliminate the data from the home address to the monitoring station greater than 50 km). Table S5. Associations between air pollutants (every 10 μg/m3 increase in the later period of retention) and hospitalization in children with bronchiolitis (Eliminate the data from the home address to the monitoring station greater than 50 km. [file 12940_2023_962_MOESM1_ESM.docx]

Supporting information for

**Short-term effects and economic burden of air pollutants on acute lower respiratory tract infections in children in Southwest China: a time-series study**

Yi He^1^, Wanyanhan Jiang^1^, Xi Gao^1^, Chengwei Lin^1^, Jia Li^2^, Lian Yang^1*^

^1^ HEOA Group, School of Public Health, Chengdu University of Traditional Chinese Medicine, No. 1166 Liutai Road, Chengdu, China

^2^ HEOA Group, School of Management, Chengdu University of Traditional Chinese Medicine, No. 1166 Liutai Road, Chengdu, China

* Corresponding author: Lian Yang

E-mail: [yyanglian@163.com](mailto:yyanglian@163.com)

**Table S1.** Pearson correlation coefficients between daily meteorological factors and air pollutants for Sichuan (2017–2018)

|  | PM_2.5_ | PM_10_ | SO_2_ | NO_2_ | Temperature | Humidity |
| --- | --- | --- | --- | --- | --- | --- |
| PM_2.5_ | 1 | - | - | - | - | - |
| PM_10_ | 0.9442* | 1 | - | - | - | - |
| SO_2_ | 0.3465* | 0.3428* | 1 | - | - | - |
| NO_2_ | 0.6101* | 0.6392* | 0.2599* | 1 | - | - |
| Temperature | -0.4598* | -0.4370* | -0.1049* | -0.3091* | 1 | - |
| Humidity | -0.0110* | -0.1257* | -0.1207* | -0.0489* | -0.0927* | 1 |

*PM_2.5_* fine particulate matter, *PM_10_* inhalable particulate matter, *SO_2_* sulfur dioxide, *NO_2_* nitrogen dioxide. ^*^*P* < 0.05

**Table S2.** RR (95% CI) of stratified analyses for each air pollutant in the single-pollutant model

| Variables | Subgroup | PM_2.5_ | | PM_10_ | | SO_2_ | | NO_2_ | |
| --- | --- | --- | --- | --- | --- | --- | --- | --- | --- |
|  |  | *RR* | *95%CI* | *RR* | *95%CI* | *RR* | *95%CI* | *RR* | *95%CI* |
| Pneumonia |  |  |  |  |  |  |  |  |  |
| Sex | Boys | 1.0039 | 0.9976-1.0103 | 1.0165 | 1.0081-1.025 | 1.0244 | 1.0120-1.0369 | 1.0421 | 1.0079-1.0774 |
|  | Girls | 1.0100 | 1.0032-1.0169 | 1.0172 | 1.0081-1.0264 | 1.0256 | 1.0129-1.0385 | 1.0301 | 0.9980-1.0633 |
| Age | 0 - 1 | 1.0063 | 0.9999-1.0128 | 1.0119 | 1.0033-1.0206 | 1.0015 | 0.9892-1.0139 | 0.9827 | 0.9515-1.0149 |
|  | 2 - 4 | 1.0056 | 0.9978-1.0134 | 1.0203 | 1.0100-1.0307 | 1.0371 | 1.0233-1.0511 | 1.0851 | 1.0454-1.1263 |
|  | 5 - 14 | 1.0109 | 1.0011-1.0209 | 1.0231 | 1.0104-1.036 | 1.0388 | 1.0240-1.0539 | 1.0999 | 1.0528-1.1490 |
| Season | Warm season | 1.0481 | 1.0217-1.0751 | 1.0647 | 1.0290-1.1016 | 1.0121 | 0.9781-1.0472 | 1.0509 | 0.9347-1.1815 |
|  | Transition season | 1.0016 | 0.9887-1.0146 | 0.9927 | 0.9666-1.0194 | 0.9919 | 0.9716-1.0126 | 1.0062 | 0.9435-1.0731 |
|  | Cold season | 1.0057 | 0.9975-1.0139 | 0.9996 | 0.9876-1.0118 | 0.9957 | 0.9764-1.0154 | 1.0122 | 0.9676-1.0590 |
| Bronchitis |  |  |  |  |  |  |  |  |  |
| Sex | Boys | 1.0125 | 1.0003-1.0248 | 1.0158 | 1.0071-1.0245 | 1.0119 | 1.0007-1.0232 | 1.087 | 1.0434-1.1323 |
|  | Girls | 1.0134 | 0.9995-1.0274 | 1.0155 | 1.0057-1.0254 | 1.0040 | 0.9917-1.0164 | 1.1299 | 1.0820-1.1798 |
| Age | 0 - 1 | 1.0022 | 0.9885-1.0162 | 1.0054 | 0.9953-1.0156 | 0.9954 | 0.984-1.0069 | 0.9801 | 0.9359-1.0264 |
|  | 2 - 4 | 1.0181 | 1.0031-1.0333 | 1.0194 | 1.0089-1.0301 | 1.0210 | 1.0083-1.0340 | 1.1379 | 1.0868-1.1915 |
|  | 5 - 14 | 1.0174 | 0.9994-1.0357 | 1.0224 | 1.0100-1.0350 | 1.0154 | 1.0023-1.0287 | 1.1806 | 1.121-1.2434 |
| Season | Warm season | 1.1523 | 1.0433-1.2726 | 1.0899 | 1.0414-1.1405 | 0.9980 | 0.969-1.0278 | 1.2639 | 1.0676-1.4964 |
|  | Transition season | 1.0111 | 0.9613-1.0636 | 0.9797 | 0.9468-1.0137 | 0.9897 | 0.9663-1.0137 | 0.9785 | 0.8767-1.0922 |
|  | Cold season | 1.0136 | 1.0019-1.0254 | 1.0124 | 1.0033-1.0215 | 1.0020 | 0.9879-1.0163 | 1.0355 | 0.9895-1.0836 |

Based on the largest effect estimates in single-pollutant model, pneumonia PM_2.5_ is lag4, PM_10_ and SO_2_ are lag010，NO_2_ is lag07; In bronchitis, PM_2.5_, PM_10_, SO_2_ and NO_2_ are lag010.

*PM_2.5_* fine particulate matter, *PM_10_* inhalable particulate matter, *SO_2_* sulfur dioxide, *NO_2_* nitrogen dioxide, *CI* confidence interval.

**Table S3.** Association between air pollutants (10 µg/m^3^ increase ) and the daily hospitalization in children with ALRI by degrees of freedom per year

| Pollutants | Models | Pneumonia | Bronchiolitis |
| --- | --- | --- | --- |
| PM_2.5_ |  |  |  |
|  | df=5 | 1.0073(1.0004,1.0142) | 1.0074(0.9964,1.0185) |
|  | df=6 | 1.0032(0.9971,1.0093) | 1.0086(0.9982,1.0191) |
|  | df=8 | 1.0057(0.9999,1.0114) | 1.0155(1.0053,1.0258) |
|  | df=9 | 1.0058(1.0001,1.0116) | 1.0150(1.0048,1.0253) |
| PM_10_ |  |  |  |
|  | df=5 | 1.0143(1.0054,1.0232) | 1.0124(1.0046,1.0203) |
|  | df=6 | 1.0124(1.0046,1.0204) | 1.0127(1.0054,1.0201) |
|  | df=8 | 1.0134(1.0058,1.0211) | 1.0149(1.0076,1.0222) |
|  | df=9 | 1.0130(1.0053,1.0207) | 1.0150(1.0077,1.0223) |
| SO_2_ |  |  |  |
|  | df=5 | 1.0307(1.0168,1.0448) | 1.0159(1.0044,1.0275) |
|  | df=6 | 1.0236(1.0109,1.0364) | 1.0131(1.0021,1.0242) |
|  | df=8 | 1.0086(0.9956,1.0218) | 0.9967(0.9859,1.0075) |
|  | df=9 | 1.0100(0.9970,1.0232) | 0.9980(0.9870,1.0090) |
| NO_2_ |  |  |  |
|  | df=5 | 1.0084(0.9738,1.0441) | 1.1043(1.0637,1.1466) |
|  | df=6 | 1.0376(1.0063,1.0700) | 1.1089(1.0701,1.1491) |
|  | df=8 | 1.0099(0.9798,1.0410) | 1.0596(1.022,1.09860) |
|  | df=9 | 1.0109(0.9809,1.0419) | 1.0606(1.0231,1.0996) |

**Table S4.** Associations between air pollutants (every 10 µg/m^3^ increase in the later period of retention) and hospitalization in children with pneumonia （Eliminate the data from the home address to the monitoring station greater than 50 km）

| Lag day | PM_2.5_ | | PM_10_ | | SO_2_ | | NO_2_ | |
| --- | --- | --- | --- | --- | --- | --- | --- | --- |
|  | RR | 95% CI | RR | 95% CI | RR | 95% CI | RR | 95% CI |
| lag0 | 1.0022 | 0.9964-1.008 | 1.0028 | 0.9985-1.0071 | 1.0009 | 0.995-1.0068 | 1.0254 | 1.0048-1.0464 |
| lag1 | 1.0018 | 0.9959-1.0077 | 1.0028 | 0.9984-1.0072 | 1.0014 | 0.9956-1.0072 | 1.0333 | 1.0124-1.0546 |
| lag2 | 1.0012 | 0.9953-1.0072 | 1.0031 | 0.9987-1.0074 | 1.0021 | 0.9962-1.008 | 1.0305 | 1.0097-1.0518 |
| lag3 | 1.0008 | 0.9948-1.0068 | 1.0032 | 0.9988-1.0076 | 1.0035 | 0.9975-1.0095 | 1.0271 | 1.0061-1.0484 |
| lag4 | 1.0019 | 0.9959-1.0079 | 1.0045 | 1.0001-1.0088 | 1.0038 | 0.9978-1.0099 | 1.0335 | 1.0126-1.0549 |
| lag5 | 1.0004 | 0.9945-1.0063 | 1.0043 | 1.0000-1.0086 | 1.0042 | 0.9982-1.0102 | 1.0335 | 1.0128-1.0547 |
| lag6 | 1.0013 | 0.9954-1.0073 | 1.0044 | 1.0002-1.0088 | 1.0085 | 1.0026-1.0144 | 1.034 | 1.0133-1.055 |
| lag7 | 0.9982 | 0.9924-1.0041 | 1.0023 | 0.9980-1.0066 | 1.0065 | 1.0006-1.0125 | 1.0392 | 1.0185-1.0604 |
| Lag8 | 1.0063 | 1.0004-1.0122 | 1.0059 | 1.0016-1.0103 | 1.0046 | 0.9987-1.0106 | 1.0373 | 1.0167-1.0584 |
| Lag9 | 1.0052 | 0.9993-1.0111 | 1.0055 | 1.0012-1.0099 | 1.0015 | 0.9957-1.0073 | 1.03 | 1.0095-1.051 |
| Lag10 | 1.0079 | 1.002-1.0139 | 1.008 | 1.0037-1.0124 | 1.0044 | 0.9987-1.0102 | 1.0386 | 1.0182-1.0595 |
| lag01 | 1.0021 | 0.9959-1.0083 | 1.0031 | 0.9985-1.0077 | 1.0011 | 0.9946-1.0077 | 1.0336 | 1.011-1.0566 |
| lag02 | 1.0018 | 0.9953-1.0084 | 1.0034 | 0.9985-1.0082 | 1.0014 | 0.9943-1.0086 | 1.0377 | 1.0136-1.0625 |
| lag03 | 1.0017 | 0.9948-1.0086 | 1.0038 | 0.9986-1.0089 | 1.0025 | 0.9947-1.0102 | 1.0411 | 1.0154-1.0675 |
| lag04 | 1.0023 | 0.9949-1.0097 | 1.0048 | 0.9994-1.0102 | 1.004 | 0.9957-1.0124 | 1.049 | 1.0215-1.0771 |
| lag05 | 1.0026 | 0.9948-1.0105 | 1.0059 | 1.0002-1.0116 | 1.0056 | 0.9968-1.0145 | 1.0579 | 1.0287-1.0879 |
| lag06 | 1.0037 | 0.9954-1.012 | 1.0072 | 1.0012-1.0133 | 1.0089 | 0.9997-1.0182 | 1.0675 | 1.0365-1.0994 |
| lag07 | 1.0042 | 0.9954-1.0131 | 1.0083 | 1.002-1.0147 | 1.0122 | 1.0025-1.0219 | 1.0817 | 1.0487-1.1158 |
| Lag08 | 1.0066 | 0.9972-1.0161 | 1.0103 | 1.0036-1.0171 | 1.0134 | 1.0032-1.0237 | 1.0924 | 1.0573-1.1287 |
| Lag09 | 1.0093 | 0.9993-1.0194 | 1.0127 | 1.0055-1.0199 | 1.0131 | 1.0025-1.0238 | 1.1014 | 1.0642-1.1399 |
| Lag010 | 1.0132 | 1.0025-1.0241 | 1.016 | 1.0084-1.0237 | 1.0136 | 1.0026-1.0247 | 1.1127 | 1.0734-1.1536 |

**Table S5.** Associations between air pollutants (every 10 µg/m^3^ increase in the later period of retention) and hospitalization in children with bronchiolitis （Eliminate the data from the home address to the monitoring station greater than 50 km）

| Lag day | PM_2.5_ | | PM_10_ | | SO_2_ | | NO_2_ | |
| --- | --- | --- | --- | --- | --- | --- | --- | --- |
|  | RR | 95% CI | RR | 95% CI | RR | 95% CI | RR | 95% CI |
| lag0 | 1.0022 | 0.9964-1.008 | 1.0028 | 0.9985-1.0071 | 1.0009 | 0.995-1.0068 | 1.0254 | 1.0048-1.0464 |
| lag1 | 1.0018 | 0.9959-1.0077 | 1.0028 | 0.9984-1.0072 | 1.0014 | 0.9956-1.0072 | 1.0333 | 1.0124-1.0546 |
| lag2 | 1.0012 | 0.9953-1.0072 | 1.0031 | 0.9987-1.0074 | 1.0021 | 0.9962-1.008 | 1.0305 | 1.0097-1.0518 |
| lag3 | 1.0008 | 0.9948-1.0068 | 1.0032 | 0.9988-1.0076 | 1.0035 | 0.9975-1.0095 | 1.0271 | 1.0061-1.0484 |
| lag4 | 1.0019 | 0.9959-1.0079 | 1.0045 | 1.0001-1.0088 | 1.0038 | 0.9978-1.0099 | 1.0335 | 1.0126-1.0549 |
| lag5 | 1.0004 | 0.9945-1.0063 | 1.0043 | 1-1.0086 | 1.0042 | 0.9982-1.0102 | 1.0335 | 1.0128-1.0547 |
| lag6 | 1.0013 | 0.9954-1.0073 | 1.0044 | 1.0002-1.0088 | 1.0085 | 1.0026-1.0144 | 1.034 | 1.0133-1.055 |
| lag7 | 0.9982 | 0.9924-1.0041 | 1.0023 | 0.998-1.0066 | 1.0065 | 1.0006-1.0125 | 1.0392 | 1.0185-1.0604 |
| Lag8 | 1.0063 | 1.0004-1.0122 | 1.0059 | 1.0016-1.0103 | 1.0046 | 0.9987-1.0106 | 1.0373 | 1.0167-1.0584 |
| Lag9 | 1.0052 | 0.9993-1.0111 | 1.0055 | 1.0012-1.0099 | 1.0015 | 0.9957-1.0073 | 1.03 | 1.0095-1.051 |
| Lag10 | 1.0079 | 1.002-1.0139 | 1.008 | 1.0037-1.0124 | 1.0044 | 0.9987-1.0102 | 1.0386 | 1.0182-1.0595 |
| lag01 | 1.0021 | 0.9959-1.0083 | 1.0031 | 0.9985-1.0077 | 1.0011 | 0.9946-1.0077 | 1.0336 | 1.011-1.0566 |
| lag02 | 1.0018 | 0.9953-1.0084 | 1.0034 | 0.9985-1.0082 | 1.0014 | 0.9943-1.0086 | 1.0377 | 1.0136-1.0625 |
| lag03 | 1.0017 | 0.9948-1.0086 | 1.0038 | 0.9986-1.0089 | 1.0025 | 0.9947-1.0102 | 1.0411 | 1.0154-1.0675 |
| lag04 | 1.0023 | 0.9949-1.0097 | 1.0048 | 0.9994-1.0102 | 1.004 | 0.9957-1.0124 | 1.049 | 1.0215-1.0771 |
| lag05 | 1.0026 | 0.9948-1.0105 | 1.0059 | 1.0002-1.0116 | 1.0056 | 0.9968-1.0145 | 1.0579 | 1.0287-1.0879 |
| lag06 | 1.0037 | 0.9954-1.012 | 1.0072 | 1.0012-1.0133 | 1.0089 | 0.9997-1.0182 | 1.0675 | 1.0365-1.0994 |
| lag07 | 1.0042 | 0.9954-1.0131 | 1.0083 | 1.002-1.0147 | 1.0122 | 1.0025-1.0219 | 1.0817 | 1.0487-1.1158 |
| Lag08 | 1.0066 | 0.9972-1.0161 | 1.0103 | 1.0036-1.0171 | 1.0134 | 1.0032-1.0237 | 1.0924 | 1.0573-1.1287 |
| Lag09 | 1.0093 | 0.9993-1.0194 | 1.0127 | 1.0055-1.0199 | 1.0131 | 1.0025-1.0238 | 1.1014 | 1.0642-1.1399 |
| Lag010 | 1.0132 | 1.0025-1.0241 | 1.016 | 1.0084-1.0237 | 1.0136 | 1.0026-1.0247 | 1.1127 | 1.0734-1.1536 |
